# Effect of Renin-Angiotensin System Inhibition on Residual Kidney Function in Peritoneal Dialysis

**DOI:** 10.3390/medicina62020282

**Published:** 2026-01-30

**Authors:** Jing Xin Goh, Kamal Sud, Katrina Chau, Surjit Tarafdar, Elvira Dsouza, Nazim Bhimani, Ronald L. Castelino

**Affiliations:** 1 School of Pharmacy, Faculty of Medicine and Health, The University of Sydney, Sydney, NSW 2006, Australia; 2 Sydney Medical School, Faculty of Medicine and Health, The University of Sydney, Sydney, NSW 2006, Australia; 3 Nepean Kidney Research Centre, Department of Renal Medicine, Nepean Hospital, Kingswood, NSW 2747, Australia; 4 School of Medicine, Western Sydney University, Sydney, NSW 2751, Australia; 5 Department of Renal Medicine, Blacktown Hospital, Western Sydney Local Health District, Blacktown, NSW 2148, Australia; 6 Pharmacy Department, Blacktown Hospital, WSLHD, Blacktown, NSW 2148, Australia

**Keywords:** kidney failure, outcomes, peritoneal dialysis, renin-angiotensin system inhibitor, residual kidney function

## Abstract

*Background and Objectives*: Renin-angiotensin system inhibitors (RASIs) are recommended to preserve residual kidney function (RKF) in patients on peritoneal dialysis (PD); however, evidence of benefit is inconsistent. This study evaluated the effect of RASI on RKF decline among patients undergoing PD. *Materials and Methods*: We conducted a retrospective cohort study among PD patients at a large metropolitan dialysis centre in Australia. RKF was assessed using residual Kt/V and urine volume from PD adequacy tests. Time zero was PD initiation. RASI exposure was modelled as a time-dependent variable to avoid immortal-time bias. Linear mixed-effects models were fitted for each outcome, including random intercepts and slopes for time (years since PD start) with unstructured covariance. Fixed effects included time, RASI(t), time × RASI(t), age, sex, baseline RKF, PD modality, PD infection episodes, loop diuretic use, and comorbidities. *Results*: Of 307 PD patients, 231 met the inclusion criteria; 111 (48.1%) received RASI. RASI users were younger than non-users [65 years (IQR 56–74) vs. 72 years (IQR 61–77); *p* = 0.014]. Residual Kt/V declined by 0.26 units/year; RASI exposure showed no significant effect on urine volume trajectory and a borderline slower Kt/V decline (interaction β = +0.038, *p* = 0.069). Hospitalisation and PD-related infection rates were similar between groups. *Conclusions*: RASI therapy was not associated with meaningful RKF preservation in PD patients in this cohort. While earlier studies suggested renoprotective effects of RASI while on PD, our findings align with recent evidence of mixed efficacy. Larger prospective trials are needed to clarify the role of RASI in maintaining RKF and improving long-term outcomes in PD.

## 1. Introduction

Peritoneal dialysis (PD) is a kidney replacement modality offering patients with kidney failure a home-based, flexible alternative to haemodialysis (HD). It is particularly valued for its potential to preserve residual kidney function (RKF), which is associated with improved clinical outcomes, including better quality of life, volume and nutritional status and enhanced survival [1,2,3,4]. International Society for Peritoneal Dialysis (ISPD) guidelines recommend that the management of PD patients should prioritise preservation of RKF [5]. Consequently, strategies that maintain or slow the RKF decline are of paramount importance in the care of patients on PD.

Among the pharmacologic strategies aimed at preserving RKF, renin-angiotensin system inhibitors (RASIs)—including angiotensin-converting enzyme inhibitors (ACEIs) and angiotensin receptor blockers (ARBs)—are widely recognised for their nephroprotective and cardioprotective effects [6,7,8,9]. Emerging evidence has also highlighted the kidney benefits of angiotensin receptor-neprilysin inhibitors (ARNIs) in patients with heart failure and chronic kidney disease (CKD) [10,11,12].

Despite clear evidence suggesting that RASIs slow the decline of RKF by reducing intraglomerular pressure and tubulointerstitial fibrosis in patients not on dialysis [13,14], evidence for their use in patients on PD is limited by small sample sizes and focuses solely on glomerular filtration rate (GFR) and urine volume [7,15,16]. Furthermore, clinical decision-making is often complicated by guideline ambiguities, patient comorbidities and concerns over adverse effects such as hyperkalaemia and hypotension—all of which contribute to cautious prescribing practices [9,17] and variable prescription of these drugs.

This study seeks to address this gap by examining the utilisation of RASI therapy—including ACEI, ARBs and ARNIs among patients undergoing PD at a large metropolitan dialysis centre in Australia. The primary objective was to assess the effect of RASI on the decline in RKF and urine volume and to explore associated clinical outcomes such as hospitalisations and PD-related infection rates.

## 2. Materials and Methods

### 2.1. Study Design, Setting and Population

We performed a retrospective longitudinal analysis of patients undergoing PD at a large metropolitan dialysis centre in New South Wales, Australia. The PD service provides training and ongoing care for approximately 300 patients. All patients who were initiated on PD between 1 June 2014 and 1 June 2023 were eligible for inclusion. Patients who were anuric at the time of PD initiation (24-h urine volume < 100 mL [18,19]) were excluded. The study was approved by the institutional human research ethics committee [2021/PID03399].

### 2.2. Data Collection and Residual Kidney Function Assessment

Demographic (age, sex), clinical (primary kidney disease, dialysis vintage, comorbidities) and laboratory data were extracted from electronic medical records (eMR). Antihypertensive medication use was identified from prescription records documented in the eMR. To enhance data reliability, medication entries were cross-checked against physician letters and/or clinical notes by nephologists and senior nurses. The analytic dataset included repeated measurements of RKF, expressed as 24 h urine volume (mL) and residual Kt/V, following the dialysis centre’s standard protocol of six-monthly PD adequacy assessments. Time zero was PD initiation; follow-up continued until transfer to haemodialysis, transplantation, death, or study end. Reasons such as hypotension or hyperkalaemia were recorded specifically as causes for RASI discontinuation, while censoring occurred only at transfer, transplantation, death, or study end.

The primary exposure was the use of RASI, modelled as a time-dependent variable RASI(t), constructed relative to each patient’s RASI start and stop dates:

- RASI(t) = 0 before RASI initiation
- RASI(t) = 1 from RASI start date through to the date before RASI stop (if a stop date exists)
- Returns to 0 after RASI discontinuation.

RASI use was defined as documentation of an ACEi, ARB, or ARNI use during PD treatment, regardless of use prior to PD initiation. Mineralocorticoid receptor antagonists (spironolactone, eplerenone) were not included. Patients without at least two comparable RKF assessments were excluded.

Covariates included age, sex, PD modality (APD vs. CAPD), loop diuretic use, PD infection episodes, hypertension, diabetes mellitus, congestive heart failure and coronary artery disease.

### 2.3. Outcomes and Subgroup Analysis

Primary outcomes were residual Kt/V and residual urine volume. Secondary outcomes included PD-related infections and all-cause hospitalisations. To explore whether baseline RKF modified outcomes, patients were also stratified into tertiles of baseline residual Kt/V: Low (≤0.76), Mid (0.77–1.31) and High (>1.31). Within each tertile, comparisons were made between RASI users and non-users.

### 2.4. Statistical Analyses

Statistical analysis was conducted using SPSS (version 29.0.1.0, Armonk, NY, USA: IBM Corp) and Stata SE (version 14.2, College Station, TX, USA: StataCorp LLC).

The primary analysis used linear mixed-effects models to evaluate longitudinal changes in RKF outcomes (residual Kt/V and residual urine volume). Models included random intercepts and random slopes for time since PD initiation (years) at the patient level to account for within-patient correlation and heterogeneity in decline rates. Fixed effects included time, time-dependent RASI exposure, age, sex, baseline residual RKF (baseline Kt/V and urine volume), PD modality, loop diuretic use, PD infection episodes, hypertension, diabetes, coronary artery disease and heart failure. Adjusted trajectories were derived using margins and visualised with marginsplot at covariate means with 95% confidence intervals. Interaction terms were included to assess whether RASI use modified the association between time and RKF decline.

Hospitalisations and PD infection episodes were recorded over a fixed 18-month period and annualised to episodes per patient-year using a denominator of 1.5 years per participant. Crude rates were calculated as events divided by patient-years. Group comparisons used Poisson regression with exposure set to time at risk (years) and robust standard errors. Overdispersion was assessed using the likelihood-ratio test for α > 0; when present, negative binomial regression was applied as the primary model. Incidence rate ratios (IRRs) with 95% confidence intervals (CIs) were reported.

Baseline demographic and clinical characteristics were summarised descriptively. Normality was assessed using the Shapiro–Wilk test. Continuous variables are reported as mean ± standard deviation or median (interquartile range) and categorical variables as frequency (percentage). These comparisons (Student’s *t*-test, Mann–Whitney U test, Chi-square test) were exploratory and descriptive only, not used for primary inference. A two-sided *p*-value < 0.05 was considered statistically significant.

## 3. Results

Among 307 patients who initiated PD during the study period, 231 were included in the final analysis after excluding 76 patients with incomplete RKF data. Baseline demographics were largely similar between groups, except that excluded patients were younger [61 years (IQR 44–73) vs. 69 years (IQR 58–76); *p* = 0.001], had shorter PD duration [19 months (IQR 4–29) vs. 43 months (IQR 30–54); *p* = 0.007] and lower prevalence of hypertension (72.4% vs. 94.8%; *p* < 0.001) and diuretic use (17.7% vs. 46.1%; *p* < 0.001). Baseline demographic and clinical characteristics of the included cohort are summarised in Table 1.

### 3.1. Characteristics of Study Participants

The cohort comprised predominantly male patients (*n* = 157; 68%), with a median age of 69 years (IQR 58–76). RASI users were significantly younger than non-users [median age 65 (IQR 56–74) vs. 72 (IQR 61–77) years; *p* = 0.014]. The majority were treated with APD (*n* = 131; 56.7%).

Both groups had a high comorbidity burden (median 5 comorbidities, IQR 3–7), with similar distributions of comorbidities, primary kidney disease, PD modality, baseline antihypertensive use and laboratory parameters. The leading aetiologies of kidney disease were diabetes (43.7%), glomerular disease (21.2%) and hypertension (17.3%). Hypertension (94.4%) and diabetes (54.5%) were the most prevalent comorbidities.

Among RASI users, 82 (73.9%) received ARBs, 26 (23.4%) received ACEIs and 3 (2.7%) received ARNIs at baseline. The use of other antihypertensives, including beta blockers, calcium channel blockers and loop diuretics, at baseline was similar among RASI and non-RASI users.

### 3.2. RASI Use and Decline in Residual Kidney Function

**Figure 1:**
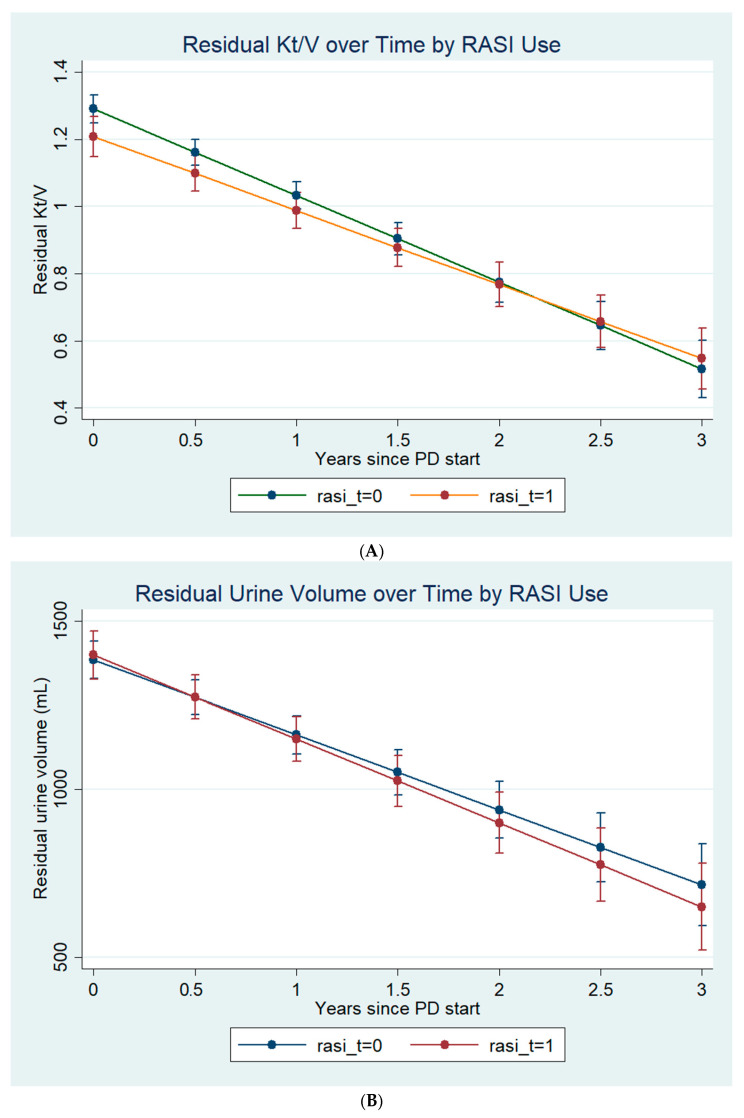
Covariate-adjusted trajectories of residual kidney function in PD patients by RASI exposure. Panels show predicted outcomes from linear mixed-effects models with random intercepts and random slopes for time (patient level). (**A**) Residual Kt/V for patients on vs. off RASI, (**B**) Residual urine volume for patients on vs. off RASI. Covariates were held at their mean values (age, gender, baseline residual RKF (baseline Kt/V and baseline urine volume), PD modality, use of loop diuretic, PD infection episodes, hypertension, diabetes, coronary artery disease and heart failure).

Each patient had a mean of 7.4 ± 3.6 PD adequacy tests. In the mixed-effects model for residual Kt/V (Table 2), time since PD start was strongly associated with decline (β = −0.258 units/year, *p* < 0.001). RASI exposure was linked to a slightly lower baseline Kt/V (β = −0.083, *p* = 0.024) and a borderline slower decline (interaction β = +0.038, *p* = 0.069). Predicted trajectories converged after ~2.2 years (Figure 1A).

For residual urine volume, decline averaged −223 mL/year (*p* < 0.001). Neither baseline effect nor interaction with time was significant for RASI exposure (*p* = 0.757 and *p* = 0.320, respectively) (Figure 1B).

**Figure 2:**
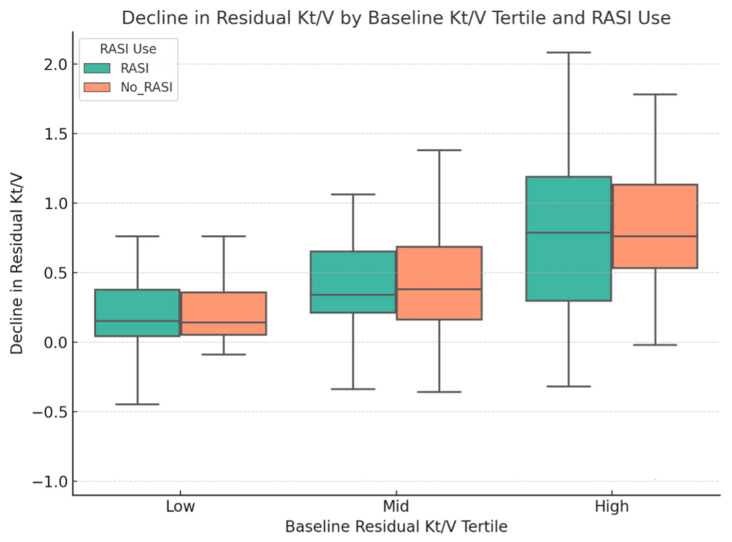
Decline in residual Kt/V across baseline residual Kt/V tertiles, stratified by RASI use.

To explore whether baseline RKF modified outcomes, patients were stratified into tertiles according to baseline residual Kt/V (Low ≤ 0.76, Mid 0.77–1.31, High > 1.31). As shown in Table 3 and Figure 2, patients with higher baseline Kt/V experienced greater absolute declines in residual Kt/V over time. Within each tertile, however, there was no significant difference in decline between patients receiving RASI and those not receiving them (interaction *p* = 0.95).

### 3.3. Hospitalisations and PD-Related Infections

Over the 18-month window, hospitalisation rates were 2.05 episodes per patient-year among RASI users and 1.77 episodes per patient-year among non-users (Table 4). Negative binomial models indicated no significant association between RASI use and hospitalisation rate (IRR 1.16, 95% CI 0.88–1.53, *p* = 0.29). PD infection rates were 0.70 versus 0.74 episodes per patient-year in RASI users and non-users, respectively; negative binomial regression again showed no significant association (IRR 0.94, 95% CI 0.66–1.34, *p* = 0.75). Model-based predicted annualised rates were similar across groups and findings were consistent in Poisson models.

## 4. Discussion

In this retrospective longitudinal study of patients undergoing PD at a large metropolitan centre in Australia, 48.1% of patients received RASI during PD treatment. Modelling RASI use as a time-dependent variable revealed no significant effect on the annual decline in residual urine volume and only borderline attenuation of residual Kt/V decline during exposure (interaction *p* = 0.069), after adjusting for demographic and clinical covariates and accounting for patient-level heterogeneity.

These findings contrast with several previous studies and meta-analyses suggesting that RASI may confer a protective effect on solute clearance and help preserve RKF in PD patients, potentially through anti-fibrotic mechanisms and haemodynamic stabilisation [7,20,21]. Such renoprotective benefits have been particularly reported with ARBs and ACEIs, which have been shown in some trials to slow the decline of RKF—a crucial determinant of outcomes including survival, quality of life and dialysis adequacy [16,22]. Several explanations may account for the divergence of higher residual urine volume and residual Kt/V in non-RASI users in our cohort. First, confounding by indication: clinicians may preferentially initiate RASIs in patients with greater comorbidity or proteinuria who are already at higher risk of RKF decline. Second, residual urine volume and residual Kt/V may also be determined by the volume status of patients affected by the use of diuretics or PD prescriptions that favour higher ultrafiltration [2,22]. Third, RKF measurement methods (e.g., eGFR vs. residual Kt/V or urine volume) and medication adherence and dosing can influence observed differences. Collectively, these factors support the interpretation that our findings likely reflect patient selection and practice heterogeneity rather than a direct detrimental effect of RASIs on RKF.

Our findings align with the recent STOP-ACEi trial and other observational studies, which found insufficient evidence supporting a benefit of RASI for the preservation of RKF in advanced CKD or dialysis populations [15,23,24]. Prior meta-analyses have also shown mixed results regarding the impact of RASIs on urine volume, progression to anuria and clinical endpoints such as cardiovascular events and mortality [7,16].

Although both ACEIs and ARBs drug classes attenuate RAAS activity, potential differences (e.g., bradykinin-mediated vasodilation/antifibrosis with ACEIs) have been hypothesised [25,26]; however, head-to-head clinical data in PD are sparse. The Cochrane review and subsequent meta-analysis suggest that either class may slow RKF decline with sufficient exposure duration, but the magnitude of benefit is small and study heterogeneity limits firm conclusions on class superiority [27,28].

When comparing these findings to data from HD populations, the role of RASI therapy appears to be more focused on reducing cardiovascular morbidity and mortality rather than preserving RKF, as most HD patients have minimal or absent RKF [29,30]. For example, Omae et al. found that RASI use in chronic HD patients was independently associated with reduced cardiovascular mortality, emphasising a distinct therapeutic goal compared to the PD population [31]. In contrast, there is sparse published data on the rate of RASI prescribing in PD populations. Although studies of broader dialysis populations report differences in prescribing patterns between HD and PD [32], RASI-specific rates are rarely disaggregated. The lower prescription rate observed in our centre compared to the prescription rate of >50% in other centres [33] may reflect concerns regarding hyperkalaemia, hypotension or clinician preference for alternative agents, but it may also suggest a potential gap in optimal cardiovascular risk management. Thus, while our analysis did not demonstrate an association between RASI use and slower decline in RKF, the broader cardioprotective benefits of RASI remain clinically important and may arguably outweigh their role in RKF preservation.

On the other hand, the absence of significant differences in hospitalisation rates and PD-related infections between RASI users and non-users in our cohort suggests that RASI therapy does not adversely affect these outcomes, supporting the overall safety profile of RASIs in the PD population. However, further prospective studies with larger sample sizes and longer follow-up are warranted to confirm these findings and explore the implications of RASI use for long-term patient outcomes.

We did not systematically evaluate mineralocorticoid receptor antagonists (MRAs) or sodium-glucose cotransporter-2 inhibitors (SGLT2i) exposure in this dataset. Emerging evidence in dialysis (HD and PD) suggests that MRAs may improve surrogate cardiac measures and, in earlier meta-analyses, were associated with lower all-cause and cardiovascular mortality [34]. However, more recent, larger meta-analyses in dialysis reports little to no effect on cardiovascular mortality after accounting for risk of bias, underscoring ongoing uncertainty and safety considerations (e.g., hyperkalaemia, gynaecomastia) [35]. With respect to SGLT2i, physiologic and preclinical rationale for benefit in PD (effects on volume status, blood pressure and peritoneal membrane biology) is compelling, but clinical evidence in PD remains limited and mixed; contemporary reviews call for adequately powered PD-specific trials, while small real-world series suggest potential short-term preservation of RKF [36,37].

### Strengths and Limitations

This is the first study from Australia to evaluate the impact of RASI therapy on the preservation of RKF and other clinical outcomes in patients undergoing PD. Unlike prior investigations that often included smaller cohorts or focused solely on GFR and urine volume, our analysis leveraged comprehensive real-world data from the largest PD centre in Australia, enabling a more representative assessment of clinical practice. Furthermore, we employed time-dependent exposure modelling and mixed-effects methods to account for repeated measures, enhancing the robustness of our findings.

Nevertheless, several limitations should be considered when interpreting these results. First, the retrospective nature of this study precludes definitive causal inference. Although adjustments were made for key confounders, residual confounding cannot be excluded. Reliance on clinical records and exclusion of patients with incomplete RKF data could have introduced selection bias. Additionally, documentation from the eMR may not accurately reflect the true duration or medication adherence over time. We acknowledge that prescription and documentation do not necessarily equate to medication consumption or adherence and patient-level compliance cannot be directly assessed. Similarly, the exact duration of RASI exposure could not be determined for all patients, although prescription records were reviewed at multiple time points to maximise accuracy. These limitations are inherent to real-world observational studies and highlight the need for prospective, adequately powered trials to more precisely delineate the benefits and risks of RASI therapy in PD patients. Additionally, the urine albumin–creatinine ratio (uACR) was not consistently documented and therefore could not be included in the analysis. Other factors such as RASI dose, ultrafiltration volume, the effect of loop diuretics on urine output and the use of biocompatible dialysate fluids were also not evaluated.

## 5. Conclusions

This study underscores that RASI therapy was not associated with meaningful preservation of RKF in patients undergoing PD within our cohort. While previous research and meta-analyses have suggested potential renoprotective benefits of ACE inhibitors and ARBs in this population, our findings align with recent large-scale trials reporting mixed or limited efficacy of RASIs for RKF preservation. Nonetheless, RASI use appeared safe, with no increase in adverse outcomes such as hospitalisation or PD-related infections. Given the complex interplay of factors influencing RKF decline and the heterogeneity of existing evidence, prospective randomised trials with longer follow-up are warranted to definitively establish the role of RASI therapy in preserving RKF and improving cardiovascular and other clinical outcomes in patients on PD.

## Figures and Tables

**Table 1:** Study characteristics based on participants prescribed with RASI.

Variables	Included Patients (*n* = 231)	Patients on RAS Inhibitor (*n* = 111)	Patients Not on RAS Inhibitor (*n* = 120)	*p* Value
*Demographics*				
**Median age, years (IQR)**	69 (58–76)	65 (56–74)	72 (61–77)	0.014 *
**Male gender, *n* (%)**	157 (68.0)	79 (71.2)	78 (65.0)	0.315
**Primary kidney disease, *n* (%)**				0.064
** Diabetic kidney disease**	101 (43.7)	53 (47.7)	48 (40.0)	
** Glomerular disease**	49 (21.2)	30 (27.0)	19 (15.8)	
** Hypertension**	40 (17.3)	14 (12.6)	26 (21.7)	
** Polycystic disease**	9 (3.9)	4 (3.6)	5 (4.2)	
**No. of comorbid conditions ^a^, median (IQR)**	5 (3–7)	5 (3–6)	5 (3–7)	0.401
**Common comorbidities ^a^, *n* (%)**				
** Hypertension**	218 (94.4)	108 (97.3)	110 (91.7)	0.064
** Diabetes mellitus**	126 (54.5)	65 (58.6)	61 (50.8)	0.239
** Atherosclerotic disease**	49 (21.2)	24 (21.6)	25 (20.8)	0.884
** Congestive heart failure**	17 (7.4)	8 (7.2)	9 (7.5)	0.932
*Dialysis characteristics*				
**PD modality, *n* (%)**				0.061
** APD**	131 (56.7)	70 (63.1)	61 (50.8)	
** CAPD**	100 (43.3)	41 (36.9)	59 (49.2)	
**PD vintage (months), median (IQR)**	43 (30–54)	43 (29–55)	42 (30–53)	0.552
**PD fluids, *n* (%)**				
** Icodextrin**	44 (19.0)	24 (21.6)	20 (16.7)	0.338
** Low GDP**	23 (10.0)	12 (10.8)	11 (9.2)	0.677
*Baseline medication use*				
**ACEi or ARB, *n* (%)**				
** ACEi**	26 (11.3)	26 (23.4)	0	
** ARB**	82 (35.5)	82 (73.9)	0	
** ARNI/ARB**	3 (1.3)	3 (2.7)	0	
**Beta blocker, *n* (%)**	111 (48.1)	51 (45.9)	60 (50.0)	0.538
**Calcium channel blocker, *n* (%)**	174 (75.3)	90 (81.1)	84 (70.0)	0.051
**Loop diuretic, *n* (%)**	100 (43.3)	51 (45.9)	49 (40.8)	0.433
*Baseline laboratory measurements*				
**Potassium (mmol/L), mean ± SD**	4.5 (0.6)	4.5 (0.6)	4.5 (0.6)	0.483
**Haemoglobin (g/L), mean ± SD**	101.5 (17.5)	102.6 (15.7)	100.4 (19.0)	0.349
**Albumin (g/L), median (IQR)**	33 (29–35)	33 (30–36)	32 (29–35)	0.272
**eGFR ^b^ (mL/min), median (IQR)**	7 (6–9)	7 (5–9)	8 (6–9)	0.136

^a^ Comorbid conditions at PD initiation. ^b^ Baseline eGFR at PD initiation. Abbreviations: ACEi, angiotensin-converting enzyme inhibitor; APD, automated peritoneal dialysis; ARB, angiotensin II receptor blocker; ARNI, angiotensin receptor-neprilysin inhibitor; CAPD, continuous ambulatory peritoneal dialysis; eGFR, estimated glomerular filtration rate; GDP, glucose degradation product; IQR, interquartile range; RAS, renin-angiotensin system; SD, standard deviation. * Statistical significance.

**Table 2:** (**a**) Association between RASI use and decline in residual Kt/V, modelled as a time-dependent variable from a mixed-effects model. (**b**) Association between RASI use and decline in residual urine volume, modelled as a time-dependent variable from a mixed-effects model.

(**a**)				
**Predictor**	**β**	**Standard** **Error**	**95% CI**	***p* Value**
**Time since PD start (years)**	−0.2583	0.0157	−0.2892 to −0.2274	<0.001 *
**RASI(t)**	−0.0826	0.0365	−0.1542 to −0.0111	0.024 *
**Time × RASI(t)**	0.0378	0.0208	−0.0030 to 0.0787	0.069
(**b**)				
**Predictor**	**β**	**Standard** **Error**	**95% CI**	***p* Value**
**Time since PD start (years)**	−223.17	22.25	−266.78 to −179.55	<0.001 *
**RASI(t)**	+14.03	45.32	−74.79 to 102.86	0.757
**Time × RASI(t)**	−26.42	26.59	−78.55 to 25.70	0.320

(**a**) Random effects: Var(intercept) = 0.0204; Var(slope) = 0.0171; Cov = 0.0067; Residual var = 0.1223; Abbreviations: CI, confidence interval; RASI, renin-angiotensin system inhibitor; * Statistical significance. (**b**) Random effects: Var(intercept) = 27,406; Var(slope) = 46,606; Cov = 14,795; Residual var = 119,690; Abbreviations: CI, confidence interval; RASI, renin-angiotensin system inhibitor; * Statistical significance.

**Table 3:** Decline in residual Kt/V over 18-month median time interval stratified by baseline residual Kt/V tertiles and RASI use.

Baseline Residual Kt/V Tertiles	Mean Decline in Residual Kt/V Over 18-Month Median Time Interval, Mean ± SD		*p* Value
	Patients on RAS Inhibitors	Patients Not on RAS Inhibitors	
**Low (≤0.76)**	0.16 ± 0.29	0.20 ± 0.25	0.58
**Mid (0.77–1.31)**	0.42 ± 0.37	0.41 ± 0.41	0.91
**High (>1.31)**	0.76 ± 0.62	0.78 ± 0.54	0.87

*p*-values derived from two-way ANOVA across tertiles. No significant interaction between tertile and RASI use (*p* = 0.95). Abbreviations: RAS, renin-angiotensin system; SD, standard deviation.

**Table 4:** Annualised hospitalisation and PD-related infection rates by RASI use.

Outcomes	Patients on RASI (*n* = 111)	Patients Not on RASI (*n* = 120)	IRR (RASI vs. Non-RASI)	95% CI	*p* Value
**No. of hospitalisation(s) (per year)**	2.054	1.767	1.163	0.881–1.534	0.287
**No. of PD infection episode(s) ^a^ (per year)**	0.703	0.744	0.944	0.663–1.344	0.749

^a^ Includes peritonitis, exit site and tunnel infections; Abbreviations: CI, confidence interval; IRR, incidence rate ratio; PD, peritoneal dialysis; RASI, renin-angiotensin system inhibitor.

## Data Availability

Data available on request from the authors.

## Abbreviations

The following abbreviations are used in this manuscript:

ACEi	Angiotensin-converting enzyme inhibitor
ARB	Angiotensin receptor blocker
ARNI	Angiotensin receptor-neprilysin inhibitor
CKD	Chronic kidney disease
eMR	Electronic medical records
GFR	Glomerular filtration rate
HD	Haemodialysis
ISPD	International Society for Peritoneal Dialysis
PD	Peritoneal dialysis
RASI	Renin-angiotensin system inhibitors
RKF	Residual kidney function
